# Genetic association between PSA-158G/A polymorphism and the susceptibility of benign prostatic hyperplasia: a meta-analysis

**DOI:** 10.18632/oncotarget.15424

**Published:** 2017-02-16

**Authors:** Xin-Jun Su, Xian-Tao Zeng, Cheng Fang, Tong-Zu Liu, Xing-Huan Wang

**Affiliations:** ^1^ Department of Urology, Zhongnan Hospital of Wuhan University, Wuhan, China; ^2^ Center for Evidence-Based and Translational Medicine, Zhongnan Hospital of Wuhan University, Wuhan, China; ^3^ Department of Evidence-Based Medicine and Clinical Epidemiology, The Second Clinical College, Wuhan University, Wuhan, China

**Keywords:** PSA, polymorphism, BPH, meta-analysis

## Abstract

Inconsistency between reported findings on the association of prostate specific antigen (*PSA*) gene -158G/A polymorphism with benign prostatic hyperplasia (BPH) susceptibility need a meta-analysis to obtain a more accurate conclusion. A systematic search was conducted in electronic databases for the collection of eligible studies on *PSA* -158G/A polymorphism and BPH susceptibility. Pooled odds ratios (ORs) and 95% confidence intervals (95% CIs) were then calculated. 7 case-control studies with 758 cases and 752 controls were included into the present meta-analysis. The analysis results showed no significant relationship between *PSA* -158G/A polymorphism and BPH susceptibility in total analysis. Interestingly, after subgroup analyses based on ethnicity and source of control, the polymorphism reduced the susceptibility of BPH in Caucasian group (AA *vs*. GG: OR=0.47, 95% CI=0.25-0.89; allele A *vs*. allele G: OR=0.68, 95% CI=0.49-0.93), but it increased the disease susceptibility in Asian (AA *vs*. GG: OR=1.63, 95% CI=1.02-2.60; allele A *vs*. allele G: OR=1.37, 95% CI=1.03-1.83) and population-based (AA *vs*. GG: OR=2.39, 95% CI=1.07-5.38; allele A *vs*. allele G: OR=1.83, 95% CI=1.26-2.65) groups. *PSA*-158G/A polymorphism may be an inhibitor to the incidence of BPH in Caucasians, but it is likely to be a susceptible factor in Asians.

## INTRODUCTION

Prostate glands are the largest accessory glands in male genital organs, with pivotal physiological functions. Benign prostatic hyperplasia (BPH) is the most common disease causing urination disorder in medium-elderly males, and its morbidity is increasing with age [1–2]. BPH usually occurs in people beyond the age of 40, and its incidence rate can reach 50% and 83% in people aged 60 and 80 years, respectively [3–4]. According to statistics, approximately half of the BPH patients will undergo moderate or severe lower urinary tract symptom (LUTS) [5] which gravely impacts their living quality. At present, a few factors correlated with BPH clinical progression have been identified, including age, serum prostate specific antigen (PSA), prostate volume, maximum flow rate, postvoid residual urine (PVRV) and international prostate symptom score (I-PSS) [6–7]. Along with the advancement of population aging, BPH has been becoming more prevalent and inducing a series of medical and social-economic problems.

Prostate specific antigen (PSA) is a glucoproteinase, and its active form is a serine protease composed by 237 residues [8]. 70% of PSA have chymotrypsin-like activity, and are closely related to male fertility [9]. In addition, PSA can hydrolyze insulin-like growth factor binding proteins for the release of active free insulin-like growth factors (IGF-Is) which are secreted by epithelial cells and can promote cell's growth through paracrine [10]. Since IGF-I receptors are distributed on the surfaces of both normal and malignant prostatic cells, it has been hypothesized that PSA possesses certain pathological effects on the growth of prostate cancer cells [11]. It has been confirmed that the expression of PSA is regulated by androgen [12], as well as by its coding gene. The gene *PSA* is located on the long arm of chromosome 19, and contains 5 exons, 4 introns and 3 promoters. Documents have demonstrated that the polymorphism-158G/A in this gene can potentially affect the transcriptional control of androgen over expression, and thus exert certain influences on PSA expression and prostate development [13–14].

Association researches have previously discussed the effect of *PSA* gene -158G/A polymorphism on BPH risk, but the findings remained contradictory. Consequently, present meta-analysis was carried out backed by all available studies to systematically clarify such relationship.

## RESULTS

### Study characteristics

**Figure 1:**
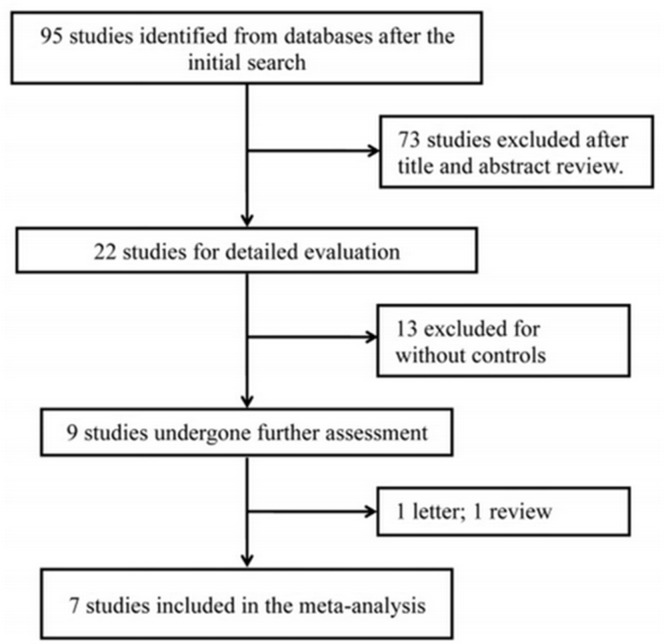
Flow diagram of selecting eligible studies for the meta-analysis

As presented in Figure 1, 95 studies were retrieved from databases after the initial search, and 73 of them were excluded after title and abstract review. Among the rest of 22 studies, 15 more were removed due to letter (*n* = 1), review (*n* = 1) and without controls (*n* = 13). Therefore, a total of 7 independent studies [15–21] were ultimately included in this meta-analysis, containing 758 cases and 752 controls. The principal characteristics of included studies are listed in Table 1. Genotype distribution in controls was corresponded to HWE in all but one study [18].

**Table 1:** Principal characteristics of the studies included in the meta-analysis

First author-Year	Country	Ethnicity	Control source	Genotyping method	Case			Control			HWE
					GG	GA	AA	GG	GA	AA	
Alptekin-2012	Turkey	Caucasian	Population-based	PCR-RFLP	2	6	3	1	9	1	0.035
Ersekerci-2015	Turkey	Caucasian	Hospital-based	PCR-RFLP	8	13	7	7	13	10	0.495
Gunes-2007	Turkey	Caucasian	Hospital-based	PCR-RFLP	34	62	40	15	44	43	0.499
Sobti-2008	India	Asian	Hospital-based	PCR-RFLP	57	99	14	74	84	12	0.068
Soni-2012	India	Asian	Population-based	PCR-RFLP	32	70	18	57	36	13	0.065
Wang-2003	Japan	Asian	Hospital-based	PCR-RFLP	125	77	14	163	88	15	0.497
Binnie-2005	UK	Caucasian	Population-based	PCR-RFLP	14	63		11	56		NA

PCR-RFLP, polymerase chain reaction-restriction fragment length polymorphism; HWE, Hardy-Weinberg equilibrium; NA, not available.

### Meta-analysis results

**Figure 2:**
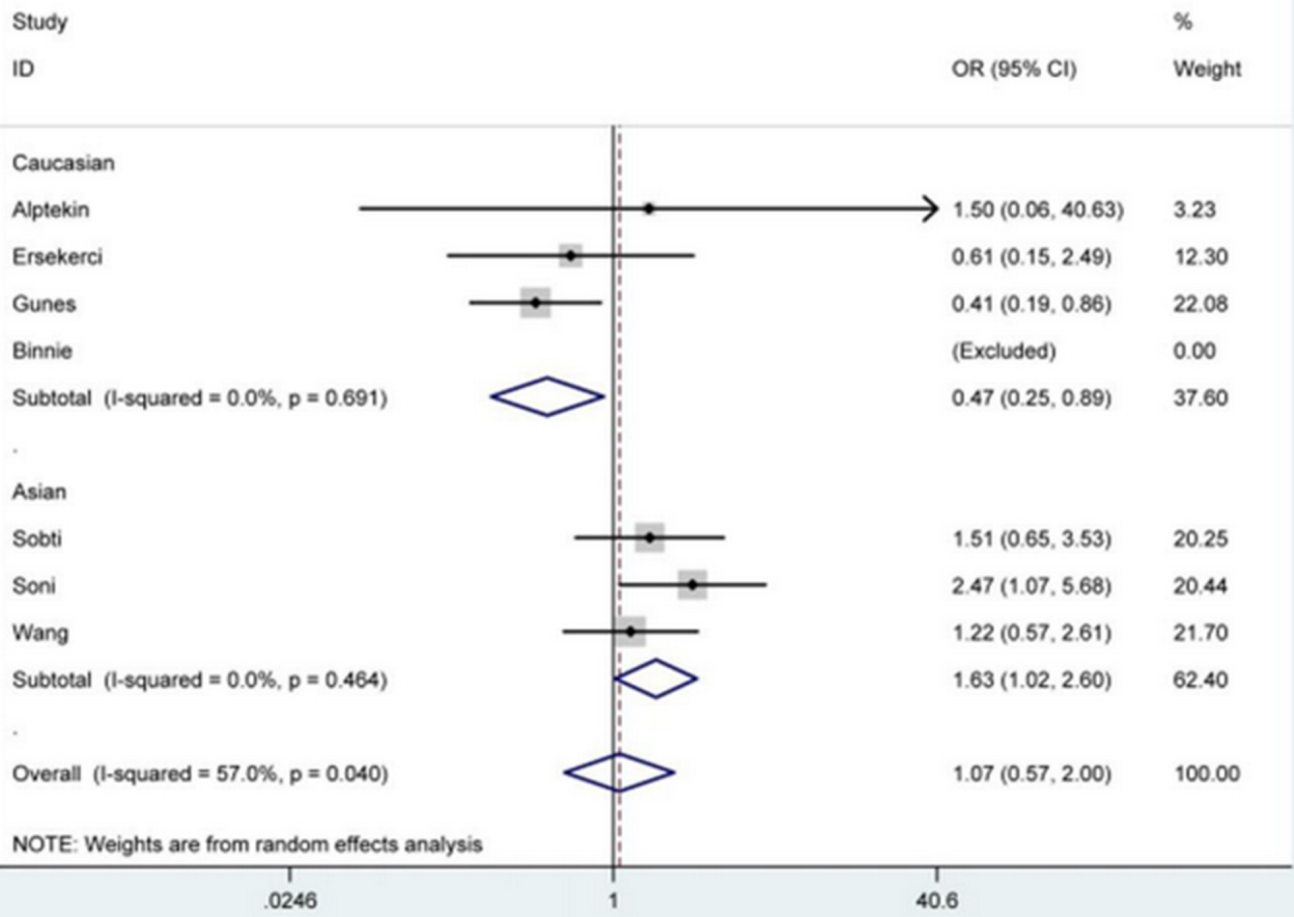
Forest plot of BPH susceptibility associated with PSA -158G/A polymorphism under AA ***vs***. GG model after stratification analysis by ethnicity. The squares and horizontal lines correspond to the study-specific OR and 95% CI. The area of the squares reflects the weight (inverse of the variance). The diamond represents the summary OR and 95% CI.

**Figure 3:**
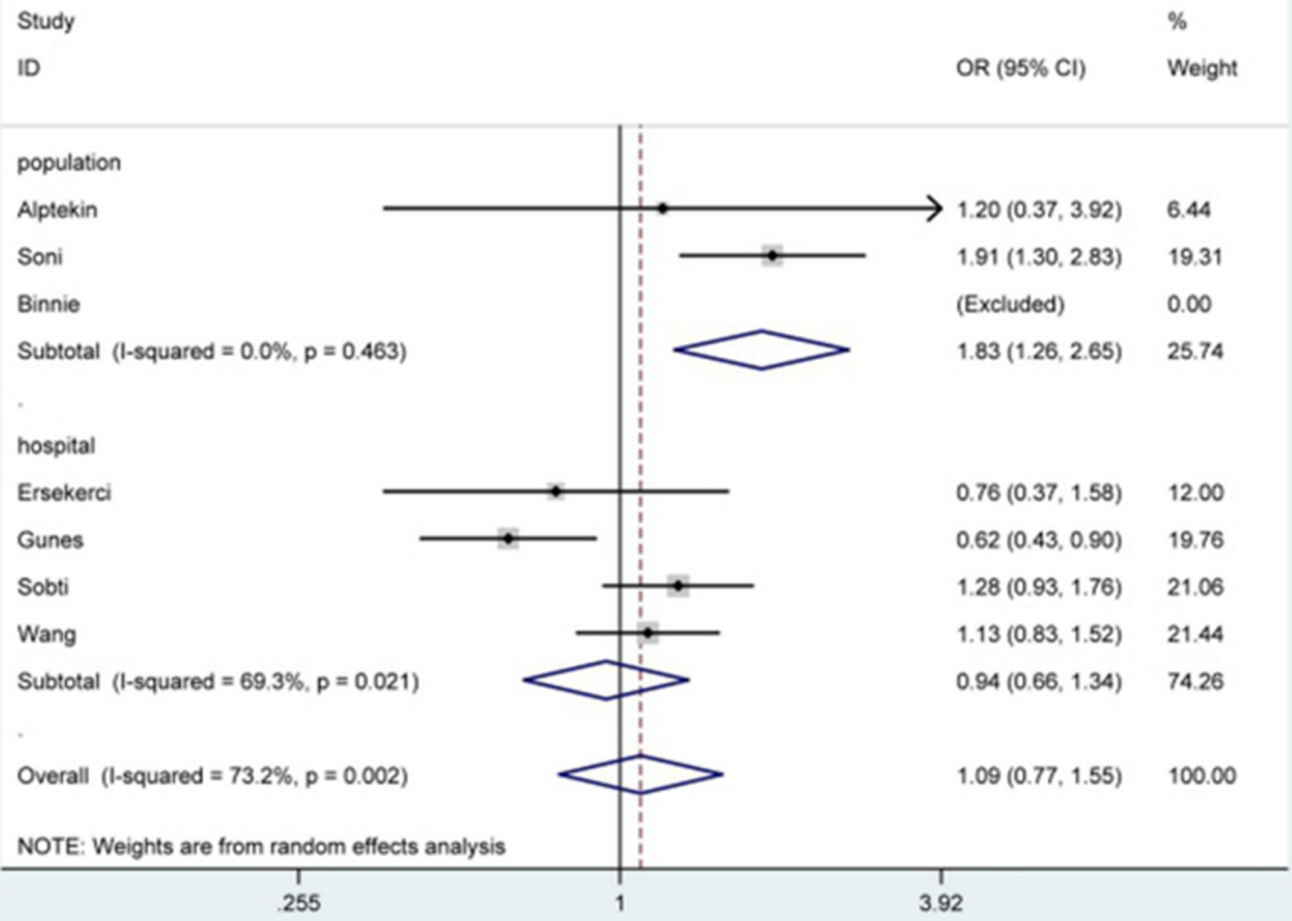
Forest plot of BPH susceptibility associated with PSA -158G/A polymorphism under allele A ***vs***. allele G model after stratification analysis by source of control. The squares and horizontal lines correspond to the study-specific OR and 95% CI. The area of the squares reflects the weight (inverse of the variance). The diamond represents the summary OR and 95% CI.

The meta-analysis results in Table 2 demonstrated that *PSA* -158G/A polymorphism didn't affect the susceptibility of BPH significantly under all genetic models in total analysis. However, after subgroup analyses by ethnicity and source of control, *PSA* -158G/A polymorphism was correlated with decreased susceptibility of BPH in Caucasian group [AA *vs*. GG: OR = 0.47, 95% CI = 0.25-0.89 (Figure 2); allele A *vs*. allele G: OR = 0.68, 95% CI = 0.49-0.93], and linked with increased risk in Asian [AA *vs*. GG: OR = 1.63, 95% CI = 1.02-2.60 (Figure 2); allele A *vs*. allele G: OR = 1.37, 95% CI = 1.03-1.83] and population-based [AA *vs*. GG: OR = 2.39, 95% CI = 1.07-5.38; allele A *vs*. allele G: OR = 1.83, 95% CI = 1.26-2.65 (Figure 3)] groups.

**Table 2:** *PSA*-158G/A polymorphism and the susceptibility to benign prostatic hyperplasia

Genetic comparison	Group/Subgroup		OR (95%CI)	*P*^h^
AA vs.GG	Ethnicity	Caucasian	0.47 (0.25, 0.89)	0.691
		Asian	1.63 (1.02, 2.60)	0.464
	Source of control	Population	2.39 (1.07, 5.38)	0.775
		Hospital	0.84 (0.44, 1.63)	0.089
		Total	1.07 (0.57, 2.00)	0.040
AA+GA vs. GG	Ethnicity	Caucasian	0.64 (0.40, 1.04)	0.784
		Asian	1.72 (0.99, 2.99)	0.011
	Source of control	Population	1.45 (0.46, 4.55)	0.025
		Hospital	1.00 (0.64, 1.58)	0.059
		Total	1.16 (0.72, 1.86)	0.002
AA vs. GG+GA	Ethnicity	Caucasian	0.64 (0.40, 1.03)	0.336
		Asian	1.20 (0.77, 1.88)	0.987
	Source of control	Population	1.41 (0.68, 2.91)	0.405
		Hospital	0.80 (0.56, 1.15)	0.329
		Total	0.90 (0.65, 1.24)	0.314
A vs. G	Ethnicity	Caucasian	0.68 (0.49, 0.93)	0.549
		Asian	1.37 (1.03, 1.83)	0.102
	Source of control	Population	1.83 (1.26, 2.65)	0.463
		Hospital	0.94 (0.66, 1.34)	0.021
		Total	1.09 (0.77, 1.55)	0.002
GA vs. GG	Ethnicity	Caucasian	0.65 (0.35, 1.20)	0.789
		Asian	1.76 (0.97, 3.18)	0.008
	Source of control	Population	1.54 (0.17, 13.70)	0.086
		Hospital	1.11 (0.77, 1.58)	0.211
		Total	1.28 (0.76, 2.15)	0.005

OR, odds ratio; CI, confidence interval; *P*_h_*, P*-value of heterogeneity test.

### Heterogeneity analysis

In Q test, significant heterogeneity was detected in AA *vs*. GG model (*P* = 0.040), AA+GA *vs*. GG model (*P* = 0.002), allele A *vs*. allele G model (*P* = 0.002) and GA *vs*. GG model (*P* = 0.005). Thus, the random-effects model was selected to evaluate overall results in these four comparisons, while the fixed-effects model was used for the other model (*P* = 0.314). As for the significant heterogeneity, it was dramatically attenuated or completely removed after stratification analyses by ethnicity and control source. We therefore hypothesized that its plausible origins might include these two aspects.

### Sensitivity analysis

We omitted individual studies one at a time and re-calculated overall estimates to detect the impact of each included study on pooled results. Since pooled ORs were not substantially altered, our results were statistically stable and credible.

### Evaluation of publication bias

**Figure 4:**
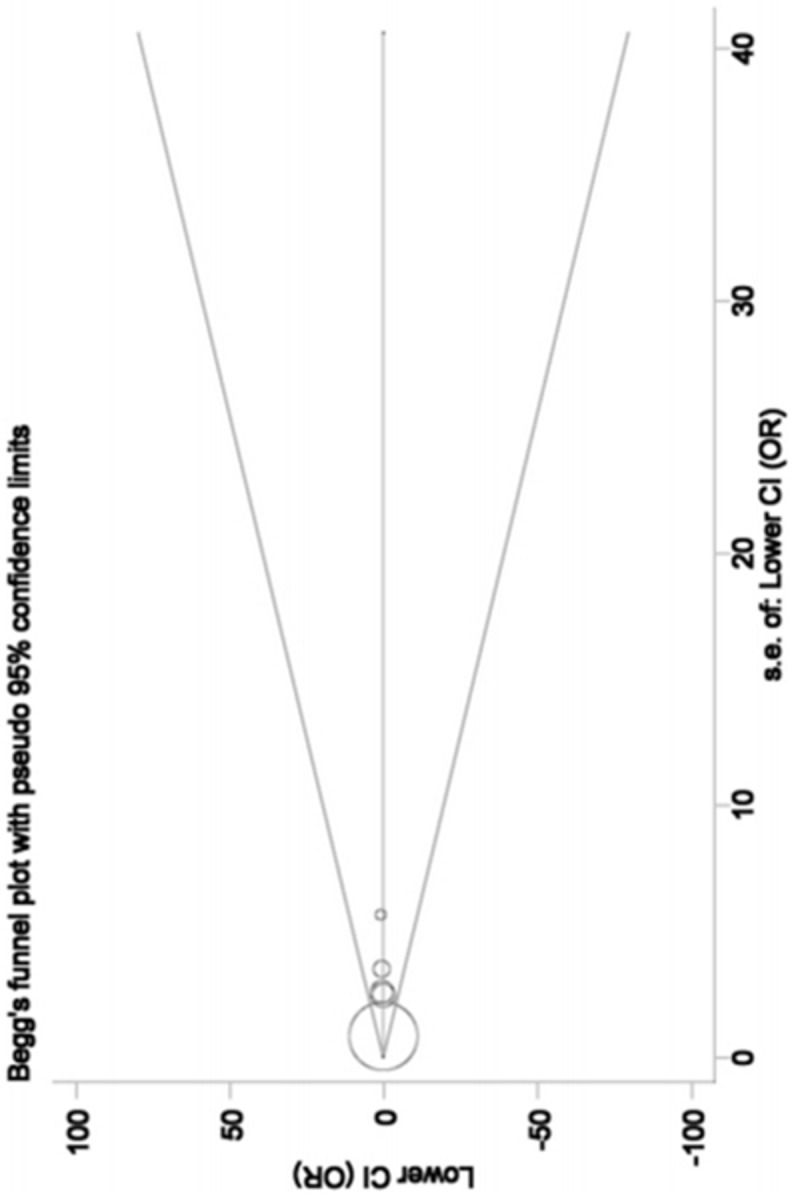
Begg's funnel plot for publication bias under the model AA ***vs***. GG. Each point represents a separate study for the indicated association. Log(OR), natural logarithm of OR. Horizontal line, mean effect size.

Both Begg's funnel plot and Egger's test were utilized to assess the possibility of existing significant publication bias. All funnel plots displayed symmetrical shapes (Figure 4), and Egger's test also provided evidence for the symmetry (*P* = 0.150), indicating publication bias was negligible.

## DISSCUSSION

BPH is a chronic progression disease, involving multiple factors and bothering elderly men in general [22–23]. Multiple hypotheses have been put forward for the pathogenesis of BPH, involving effects of hormone and intratesticular non-androgen materials, apoptosis, mesenchyme-epithelium interaction and growth factors. Literature has shown that age and testosterone level could impact the incidence of BPH notably. In addition, different races and genetic backgrounds are also considered as relevant components to BPH development.

PSA, regulated by androgen, is produced by prostate cells, and is generally used as a prostate disease marker to predict the enlargement of prostate volume clinically. *PSA* gene is polymorphic, and among others, a polymorphism at position -158 (G/A) is one of the most frequently studied objects. In a Turkey population, Gunes et al. found an increasing effect of the GG genotype of *PSA* -158G/A polymorphism on BPH susceptibility, with an age-adjusted OR of 2.77 (95% CI = 1.277-6.012) [20]. On the contrary, Abha Soni et al. insisted that the genotypes AA and GA of this polymorphism were significantly related to elevated risk of BPH in their study involving 120 BPH cases and 105 controls in Indians [21]. Whereas, a study by Alptekin and colleagues detected no statistically significant difference in the frequencies of genotypes or alleles of *PSA* -158G/A polymorphism between controls and BPH cases in Turks, suggesting that the polymorphism might not have independent influence on the disease onset [18].

It is necessary to perform a meta-analysis so as to combine those conflicting findings on the relationship between *PSA* -158G/A polymorphism and BPH susceptibility for a more convincing conclusion in view of distinct discrepancies among those findings. In our study, *PSA* -158G/A polymorphism showed no significant impact on BPH incidence in total analysis, but we observed with great interest that this polymorphism exerted opposite effects in different subgroups after stratification analysis by ethnicity and source of control. Specifically, under the genetic comparisons of AA *vs*. GG and A *vs*. G, *PSA* -158G/A polymorphism reduced the disease risk in Caucasian group, and increased the risk in Asian and population-based groups. To verify the reliability of the overall estimates from the present meta-analysis, we also implemented a series of tests. In sensitivity analysis, no qualitative alteration in pooled ORs was observed after the omission of any one of included studies, confirming the robustness of final outcomes. Besides, both funnel plot and Egger's test offered evidences supporting the absence of significant publication bias among selected studies. Hence, our study results had certain powerful in themselves.

Nevertheless, several limitations still needed to be acknowledged. First of all, we only included the articles previously published through online database searching, so possibly relevant reports unpublished or in other sources might be missed. Secondly, only seven eligible studies were ultimately incorporated into the current meta-analysis, with a relatively small sample size, and this situation might affect the precision of our results. Thirdly, not all eligible provided data adjusted for potentially confounding factors, which might introduce some bias into the final results. Fourthly, effects of gene-environment and gene-gene interactions on BPH susceptibility were not considered in this paper owing to inadequate data. Finally, since meta-analysis is a secondary analysis, the overall estimates may be affected by the quality of included studies. Therefore, our findings should be interpreted prudently.

In short, this meta-analysis suggests that *PSA* -158G/A polymorphism may be a protecting factor against BPH in Caucasian populations, but it may enhance the disease risk in Asians. In view of the shortcomings mentioned above, well-designed studies with larger sample sizes are warranted to verify these findings in future.

## MATERIALS AND METHODS

### Inclusion and exclusion criteria

Studies were included into this meta-analysis if they met the following criteria: (i) exploring the correlation of *PSA* gene -158G/A polymorphism with BPH susceptibility; (ii) case-control studies; (iii) sufficient data for computing crude odds ratios (ORs) with their 95% confidence intervals (95% CIs). Papers not satisfying any one of the above standards were excluded from the current study. In addition, removed publications also contained letters and reviews.

### Search for eligible studies

Relevant studies were searched from PubMed, EMBASE, Chinese National Knowledge Infrastructure (CNKI) and Web of Science databases. Combinations of the following terms were adopted: “prostate specific antigen” or “PSA” or “KLK3” or “-158G/A”, “polymorphism” or “mutation” or “variant”, and “benign prostatic hyperplasia” or “BPH” or “benign prostate hyperplasia”. References cited in pertinent articles were also screened manually to search for additional relevant papers.

### Data extraction

Two reviewers abstracted the following information independently from included studies: first author's name, year of publication, original country, ethnicity, control source, genotyping method, numbers of cases and controls, genotype frequencies in case and control groups, and *P* values for Hardy-Weinberg equilibrium (HWE) in controls. All discrepancies over extracted data were resolved *via* discussion between the two reviewers. If not, the third reviewer would be consulted to reach a final determination.

### Statistical analysis

All the statistical tests were conducted using the STATA (version 12.0; StataCorp, College Station, TX) software. The conformity of genotype distribution to HWE in controls was investigated through the goodness of fit chi-square test, and *P* > 0.05 were regarded as fine compliance to the Law. Pooled ORs with 95% CIs were calculated to estimate the relationship between *PSA* -158G/A polymorphism and BPH susceptibility under five models: AA *vs*. GG, AA+GA *vs*. GG, AA *vs*. GG+GA, allele A *vs*. allele G and GA *vs*. GG. The significance of the pooled ORs was determined by Z-test. Subgroup analyses according to ethnicity and source of control were performed so as to further test such relationship. Heterogeneity among the included studies was checked by chi-square based Q-test, and *P* > 0.05 in the test suggesting the absence of significant heterogeneity among studies determined that the pooled ORs were computed with fixed-effects model [24]. Otherwise, random-effects model was used (*P* < 0.05) [25]. Sensitivity analysis was implemented to examine the influence of each single study on pooled results. Begg's funnel plots and Egger's test [26] were used to measure potential publication bias across eligible studies.
